# Tissue-specific transcriptome analysis of drought stress and rehydration in *Trachycarpus fortunei* at seedling

**DOI:** 10.7717/peerj.10933

**Published:** 2021-04-01

**Authors:** Xiao Feng, Zhao Yang, Xiurong Wang

**Affiliations:** 1 College of Forestry, Guizhou University, Guiyang, Huaxi, China; 2 Institute for Forest Resources & Environment of Guizhou, Guizhou University, Guiyang, Huaxi, China; 3 Key laboratory of Forest Cultivation in Plateau Mountain of Guizhou Province, Guizhou University, Guiyang, Huaxi, China; 4 Key laboratory of Plant Resource Conservation and Germplasm Innovation in Mountainous Region (Ministry of Education), Guizhou University, Guiyang, Huaxi, China

**Keywords:** *Trachycarpus fortunei*, Transcriptome, Drought stress, Rehydration, WGCNA

## Abstract

**Background:**

*Trachycarpus fortunei* has broad economic benefits and excellent drought resistance; however, its drought response, adaptation, and recovery processes remain unclear.

**Methodology:**

In this study, the response, tolerance, and recovery processes of *T. fortunei* leaves and roots under drought stress were determined by Illumina sequencing.

**Results:**

Under drought stress, *T. fortunei* reduced its light-capturing ability and composition of its photosynthetic apparatus, thereby reducing photosynthesis to prevent photo-induced chloroplast reactive oxygen damage during dehydration. The phenylpropanoid biosynthesis process in the roots was suppressed, *DHNs*, *LEA, Annexin D2, NAC*, and other genes, which may play important roles in protecting the cell membrane’s permeability in *T. fortunei* root tissues. During the rehydration phase, fatty acid biosynthesis in *T. fortunei* roots was repressed. Weighted correlation network analysis (WGCNA) screened modules that were positively or negatively correlated with physiological traits. The real-time quantitative PCR (RT-qPCR) results indicated the reliability of the transcriptomic data.

**Conclusion:**

These findings provide valuable information for identifying important components in the *T. fortunei* drought signaling network and enhances our understanding of the molecular mechanisms by which *T. fortunei* responds to drought stress.

## Introduction

*Trachycarpus fortunei* (Hook.) H. Wendl. (Fam.: *Palmae*; Gen.: *Trachycarpus*) is an evergreen tree that is widely distributed. It is an important economic and landscaping plant. The global climate is warming, the land area affected by water shortage is expanding, the frequency of droughts is increasing, and the severity and duration of droughts are increasing (Stocker et al., 2014; Yao et al., 2015; Lamaoui et al., 2018). The drought stress experiment of 2-year-old *T. fortunei* from 19 provenances in Guizhou and Yunnan of China found that soluble sugar and Superoxide dismutase (SOD) reached the maximum value at 14 date, due to the difference in the ability of the provenances to resist drought, the lethality of seedlings reached 40% after rehydration of some provenances (Lan et al., 2017). However, there is still a lack of research on the response and adaptation of *T. fortunei* to drought response.

Typical mechanisms for plants to resist drought include the development of vigorous root systems, the formation of epidermal wax, a thicker epidermal layer, regulation of stomatal conductance to reduce transpiration, elimination of reactive oxygen species, and activation of stress-related hormones. Under the pressure of drought environment, the leaves will close the stomata (Hummel et al., 2010), and the cells will adjust the osmosis to increase the amount of free sugar and free amino acids (especially proline) (Showler, 2013). When it persists, the protein may lose its activity or become denatured, producing excess reactive oxygen species (ROS), stimulating the activity of oxidative stress kinases in leaf cells (Anjum et al., 2011), photosynthesis is inhibited, leading to metabolic dysfunction and destruction of cell structure (Krasensky & Jonak, 2012; Li et al., 2016). ROS are rapidly cleared due to various drought-induced signaling pathways regulated by abscisic acid (ABA) (Contreras-Porcia et al., 2011). Productive ROS signaling requires both rapid increases in ROS, as well as the ability of cells to prevent ROS from reaching damaging levels through synthesis of antioxidants, including flavonols (Postiglione & Muday, 2020). ABA is a predominant hormone that regulates stomatal closure under drought stress. Plants recognize water deficit conditions in roots and that several molecular signals then move from roots to shoots (Takahashi et al., 2020). CLAVATA3/EMBRYO-SURROUNDING REGION-RELATED 25 (CLE25) peptide transmits water-deficiency signals through vascular tissues, and affects ABA biosynthesis and stomatal control of transpiration in association with BARELY ANY MERISTEM (BAM) receptors in leaves (Takahashi et al., 2018). ABA accumulation is enhanced mainly in the vasculature of the leaves, accumulated ABA is supposed to spread from the vasculature to all tissues to mediate stomatal movements and gene expression related to drought stress resistance (Kuromori, Seo & Shinozaki, 2018; Takahashi et al., 2020), drought-responsive genes mainly involved photosynthesis, signal transduction, lipid metabolism, sugar metabolism, wax synthesis, cell wall regulation, osmotic adjustment (Chen et al., 2020). When the damage exceeds a certain level, it will cause plant plastic damage, growth retardation, or even death. So far, little is known about the regulation during the stress response process of *T. fortunei*, and the dynamic changes of genes when *T. fortunei* reaches severe drought are still unknown.

The acquisition of full-sibling progeny is to retain the excellent traits of the family through sexual crossing, with the same genetic background, eliminating the influence of different genotype differences within the species. The dynamic changes of physiological indicators are the products of differential gene expression, and the tolerance to drought is a complex phenotypic trait controlled by multiple genes. Transcriptomics analysis is used to identify genes that play an important role in drought tolerance and infer the main mechanisms involved (Li et al., 2017). At present, there is a lack of *T. fortunei* genome and transcriptome data, and relevant information is not available. In order to understand the molecular resistance and recovery mechanisms of *T. fortunei*, through the simulation of natural drought stress process at seedling stage, the expression profile changes of related resistance genes were obtained, combined with phenotypes and physiological indicators to evaluate the molecular response of *T. fortunei*.

## Materials and Methods

### Test

In April 2017, we conducted a *T. fortunei* germplasm survey and selected excellent male and female plants for hybridization, the excellent paternal pollen collected in Guiding, Guizhou province, China, was sexually crossed with the excellent female plants in Huaxi, Guizhou province, China. The full-sibling progeny were obtained. The full-sibling progeny of 1.5-year-old seedlings was selected for transplanting and placed in a greenhouse (Normal growth 4 months after transplanting). The soil type in the pot was humus:yellow soil (1:3). Natural drought stress was simulated and all plants were normally irrigated for 3 days before drought treatment. After the irrigation was stopped, sampling was conducted between 8:00–9:00 in the morning, leaves and root tips were collected every three days from new plants (some leaves were immediately frozen in liquid nitrogen for transcriptome sequencing and RT-qPCR, while others were used for biochemical parameters measurements), phenotypic changes were also recorded. The leaf collection site was unified into mature healthy leaves, root tips were collected from the roots. Samples were collected every 3 days until the fresh leaves of the seedlings were extremely atrophic. A total of 15 days between the cessation of irrigation and the end of the severe drought. At this point, drought stress concluded and the rehydration experiment was conducted, rehydration was performed on the same day to the control (0d) level. After 12 h (Re0.5d), 1 day (Re1d), 3 days (Re3d), and 6 days (Re6d), rehydration and related indicators were measured. The aluminum box soil drying method was used to determine the soil absolute moisture content (AMC). A leaf area scanner was used to measure the *T. fortunei* leaf area.

### Test methods

#### Determination of biochemical parameters

First, mature leaves and root tips were collected. Using the methods described in The Principle and Technology of Plant Physiology and Biochemistry Experiment Book (Xuekui, 2006) to determine the following indicators. The proline (Pro) content in the leafs were determined according to the method of acid ninhydrin colorimetry (Demirel et al., 2020). The level of Malondialdehyde (MDA) content in the leaves (Ml) and roots (Mr) were determined by Thiobarbituric acid (TBA) method (Liu, Wei & Li, 2014). Leaf superoxide dismutase (SOD) activity was measured spectrophotometrically at 560 nm using the inhibition of photochemical reduction of nitro-blue tetrazolium (NBT) in the presence of riboflavin under light according to Giannopolitis & Ries (1977). Root activity (Rv) in the roots was determined usingtriphenyltetrazolium chloride (TTC) method. Peroxidase (POD) activity was determined using the phenol method. The experiment included 3 biological and technical replicates. The statistics and analysis of physiological related data are perfomed using R software (https://www.r-project.org/), Cluster analysis of the samples was performed using ggtree software package (Yu et al., 2017). A principal components analysis (PCA) was performed using FactoMineR software package (Le, Josse & Husson, 2008).

#### Transcriptome material collection and library construction

The material selection time nodes included 0 d (start drought), 9 d (ninth day of drought), 15 d (15th day of drought), Re0.5d (twelfth hour after rehydration), and Re1d (the first day/24 h after rehydration), collect the roots tips and mature leaves of the above time nodes, each with three biological replicates. The leaf collection site was unified into mature healthy leaves. Root tips were collected from the roots, washed with distilled water, wrapped in tin foil, and stored in liquid nitrogen. The isolation of total RNA from above samples were isolated according to the instruction manual of the Trizol Reagent (Invitrogen, Carlsbad, CA, USA). Nandrop 2000 (Thermo Fisher Scientific, Waltham, MA, USA) was used to determine the concentration and purity of the RNA. The RNA integrity was assessed by agarose gel electrophoresis while its integrity number (RIN) value was measured by Agilent 2100 (Agilent Technologies, Santa Clara, CA, USA). After the quality of the total RNA sample met the requirements (with at least 1µg sample of concentration ≥ 50 ng/μL, OD260/280 = 1.8–2.2), the mRNA was enriched with Oligo (dT) magnetic beads. Further, the mRNA was added with fragmentation buffer and cut into short fragments. Using mRNA as templates, cDNA was reverse transcribed using six-base random primers. The double-stranded cDNA samples were purified, end-repaired, added with poly(A) tails and then ligated to the sequencing adapters to create cDNA libraries. After the libraries passed quality test, they were sent to Shanghai Ouyi Co. (Shanghai, China) for sequencing from both ends with an Illumina HiSeq X Ten machine.

#### Data processing and analysis

Raw data were quality filtered and reads with adapters or poor-quality sequences were removed using the Trimmomatic tool (Bolger, Lohse & Usadel, 2014). Assembly of the reads was performed using the Trinity (Grabherr et al., 2011) software paired-end to get the assemblage of the transcripts and unigene libraries. Diamond (Buchfink, Xie & Huson, 2015) was used to compare the unigenes to the NCBI NR, EuKaryotic Orthologous Groups (KOG), Gene Ontology (GO), Swiss-Prot, eggNOG, and Kyoto Encyclopedia of Genes and Genomes (KEGG) databases. Functional analyses were conducted using the HMMER comparison of unigenes to the Pfam database (Mistry et al., 2013). The PlantTFDB database (Jin et al., 2013) was used to identify transcription factors (TFs).

Using Bowtie2 (Langmead & Salzberg, 2012), the number of unigene reads in each sample was obtained. The expression of unigene Fragments Per Kilobase of transcript per Million Mapped reads (FPKM) was calculated using eXpress (Roberts, 2013). First, the correlation heatmap and PCA analysis were performed using the expression levels of all samples, followed by PCA analysis for different tissue samples, roots and leaves. Use DESeq (Anders & Huber, 2012) software to standardize the number of Unigene counts in each sample (basemean value is used to estimate expression), calculate the difference multiple, and use NB (negative binomial distribution test) to test the difference reads significance, and finally screen differentially expressed gene (DEG) according to the difference multiple and the difference significance test results. DEG screening threshold was set to *p* < 0.05 and |foldchange| > 2. The clusterProfiler package was used to perform GO and KEGG enrichment of the DEGs (Yu et al., 2012). GOSemSim was used to calculate the similarity between GO terms (Yu et al., 2010). GO term similarity clustering was conducted using ggtree. Calculation results were selected if *q* < 0.01 in order to avoid the very general sets and limit the annotation of >300 genes and GO terms of <100 genes. According to the *p*-value screening, the top 10 KEGG pathways were used to map multiple sets of enriched analysis bubbles. Short Time-series Expression Miner (STEM) software was used to perform the trend cluster analysis (Ernst & Bar-Joseph, 2006), which was divided into five stages according to the time of occurrence; the combination of different stages of combined genes was input and normalized. The number of models was set to 50.

Screening for the top 5,000 genes of the leaf and root gene expression matrix (background gene set for all genes) was conducted according to the median absolute deviation (MAD) method. A matrix of the relationship between gene expression and sample traits was established using the weighted co-expression network analysis (WGCNA) package (Langfelder & Horvath, 2008), which was subsequently transformed into a leader matrix to construct a joint analysis of the modules and traits.

Nine genes were selected to validate the transcriptome data using real-time quantitative PCR (RT-qPCR). SuperMix was removed and cDNA was synthesized using TransScript One-Step gDNA. The first strand of the cDNA fragment was synthesized from total RNA. RT-qPCR was performed on a real-time CFX96 Touch PCR instrument. The PCR reaction conditions were as follows: preheating at 95 °C for 30 s, 40 cycles of heat denaturation at 95 °C for 5 s, and annealing at 60 °C for 34 s. The *T. fortunei* actin gene was used as the reference gene for data standardization. Each sample was repeated three times and the relative expression levels were calculated using the 2^−ΔΔCt^ method (Schmittgen & Livak, 2008). The primer sequences used in this study are provided (Table 1).

**Table 1:** The primer sequences used in this study. PCR primer sequences.

No.	Gene_ID	Forward primer	Reverse primer	Product length(bp)	gene_name
	Reference Gene	5′ TGAATCTGGTCCATCCATTGTC 3′	5′ AGAACATACCATAACCAAGCTC 3′	60	Actin
1	TRINITY_DN53033_c0_g1_i1	5′ ACCCTCCATCTCAGCCTTCA 3′	5′ TGCCTCTGTGACCTCCTTTC 3′	146	accA
2	TRINITY_DN46177_c1_g1_i1	5′ TGTCCCTATGTGCCTAGCAGTAA 3′	5′ TCCGCATCCAACAATGTAAGAG 3′	76	Annexin D2
3	TRINITY_DN35842_c0_g1_i2	5′ GACTGCTACTACGTCCCAACCTG 3′	5′ CGACAAACCAATGGCTTCTTCA 3′	81	cytochrome P450
4	TRINITY_DN51483_c1_g1_i2	5′ ATCAGGTCACCGTCCATAGC 3′	5′ TTCTCCTTCAATCCCTTCTTTT 3′	169	Dehydrin COR410
5	TRINITY_DN52956_c0_g1_i2	5′ ATAGGATTTGGCAGATAGCATTCG 3′	5′ AACGCCTTCCATACCGCACT 3′	64	KCS-11
6	TRINITY_DN48903_c0_g2_i1	5′ ACTTGCTAAGCCAGCCATTC 3′	5′ AATCCCTTTGATGCCACTCC 3′	245	NAC 47
7	TRINITY_DN58051_c0_g2_i12	5′ TGTGGCATCGTCTCAACT 3′	5′ AATGAATGCACGGTTTGA 3′	87	P5CS
8	TRINITY_DN57760_c0_g1_i2	5′ TGCTGGAAAGAGTTAGAAGAGG 3′	5′ AGATTCGATATTATGGGTGGC 3′	62	PRODH
9	TRINITY_DN27793_c0_g1_i1	5′ ATGGCAACCTTCCACTCTTCCTG 3′	5′ ACCAACTGATCCGAGCACTCCTT 3′	116	YLS3

## Results

### Plant-related physiological and biochemical indicators

**Figure 1:**
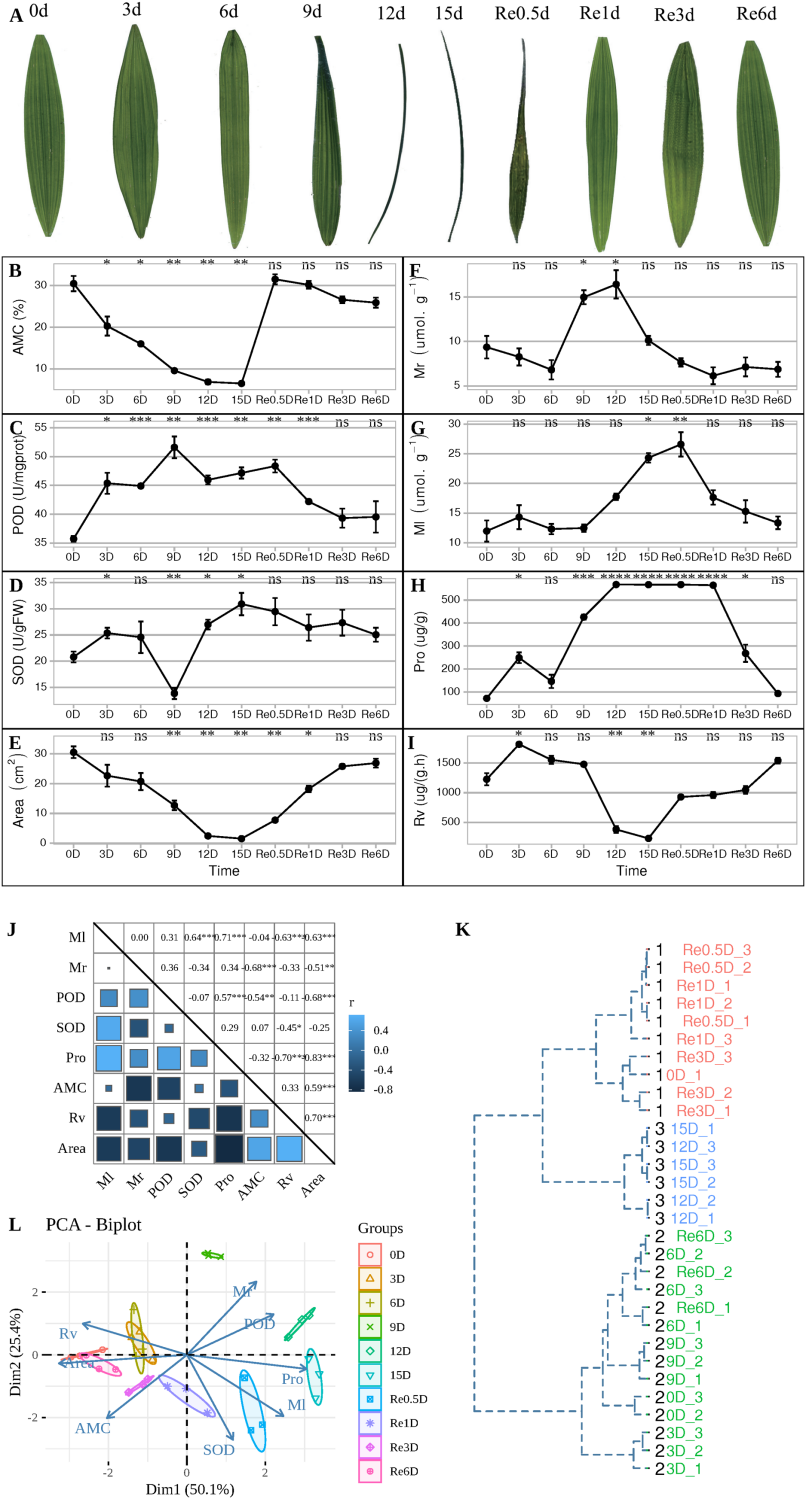
During the experiment, leaves entered the folded state after 9 d (Fig. 1A). Changes in the physiological indicators of *T. fortunei* during drought stress and rehydration. (A) Collection of *T. fortunei* leaf samples during the experiment; (B) changes in the absolute moisture content of the soil; (C) changes in the POD of *T. fortunei* leaves; (D) changes in the SOD activity of the leaves; (E) changes in the area of *T. fortunei* leaves; (F) changes in the MDA content of the roots; (G) changes in the MDA content of the roots; (H) changes in the Pro content of the leaves; (I) changes in the root activity tetrazolium reduction intensity; (J) correlation of the indices during stress; (K) cluster analysis of the samples during stress; (L) PCA of the physiological indexes during stress. In (B–I), the unpaired Welch’s *t*-test was done using the R-function *t*.test, An asterisk (*) indicates that there is difference compared with the “0d” group, *p < 0.05, **p < 0.01, ***p < 0.001, and “ns” means no difference, the error line represents the standard deviation error; the square size. In (K), the distance matrix was run with hclust, and a dendrogram was plotted that displays a hierarchical relationship among the samples. (J) Represents the correlation value, and the asterisk (*) represents the significance of correlation; members represent members grouped together.

During the experiment, leaves entered the folded state after 9 d (Fig. 1A), AMC decreased continuously from 0 to 15 d (Fig. 1B). After 9 d, the POD activity reached its maximum value (Fig. 1C), while SOD activity was at its lowest value (Fig. 1D). After 12 d, the leaves were folded and curled, leaf area decreased (Fig. 1E), and the MDA content in the roots increased to its maximum value (Fig. 1F), and the MDA content in the leaves reached its maximum value in Re0.5d (Fig. 1G), but was delayed compared to the roots. The Pro content in the leaves reached its maximum value (Fig. 1H). After 15 d, SOD activity reached its peak value, and Rv (Fig. 1I) and area decreased to their lowest values. Each indicator showed a correlation with the positive/negative modes of stress, Area was positive correlation with Rv (Correlation coefficient = 0.70, *P* = 1.918869e−05(***)), Area and Pro show negative correlation (Correlation coefficient = −0.83, *P* = 1.062141e−08(***)) (Fig. 1J). Samples of 12d and 15 d were clustered into member 2 (Fig. 1K). After rehydration, most *T. fortunei* gradually stretched at Re0.5d. PCA of physiological indexes showed that PC1 on the *X* axis captures the greatest variation and accounts for 50.1% of the total variance around the PCs whilst PC2 on the *Y* axis captures the second greatest variation and accounts for 25.4% of the total variance around the PCs (Fig. 1L), the PCA results revealed that Re6d and 0 d were closer and coercion was lifted.

### Data quality control, splicing, comparison, expression quantification, and differential analysis

The raw data of each sample (Q30) ranged from 96.88% to 97.57%. The effective data ranged from 6.98 to 7.18 G. The average GC content was 47.94%. The four base content distribution of each sample was relatively uniform. A total of 66,270 unigenes were spliced with a total length of 55,575,529 bp and an average length of 840. In total, 43,238 (65.25%) unigenes were annotated in the NCBI NR database, while 30,054 (45.35%) unigenes were annotated in the Swiss-Prot database. A total of 51,381 CDS sequences were predicted, of which, 43,373 were predicted by the database comparison method and 8,008 were predicted by ESTScan. A total of 15,696 unigene annotations were identified in the TF database and were distributed across 58 families. The raw data were stored in the NCBI/SRA database (BioProject accession No.: PRJNA598974), this transcriptome Shotgun Assembly project has been deposited at DDBJ/EMBL/GenBank under the accession GIYF00000000.

**Figure 2:**
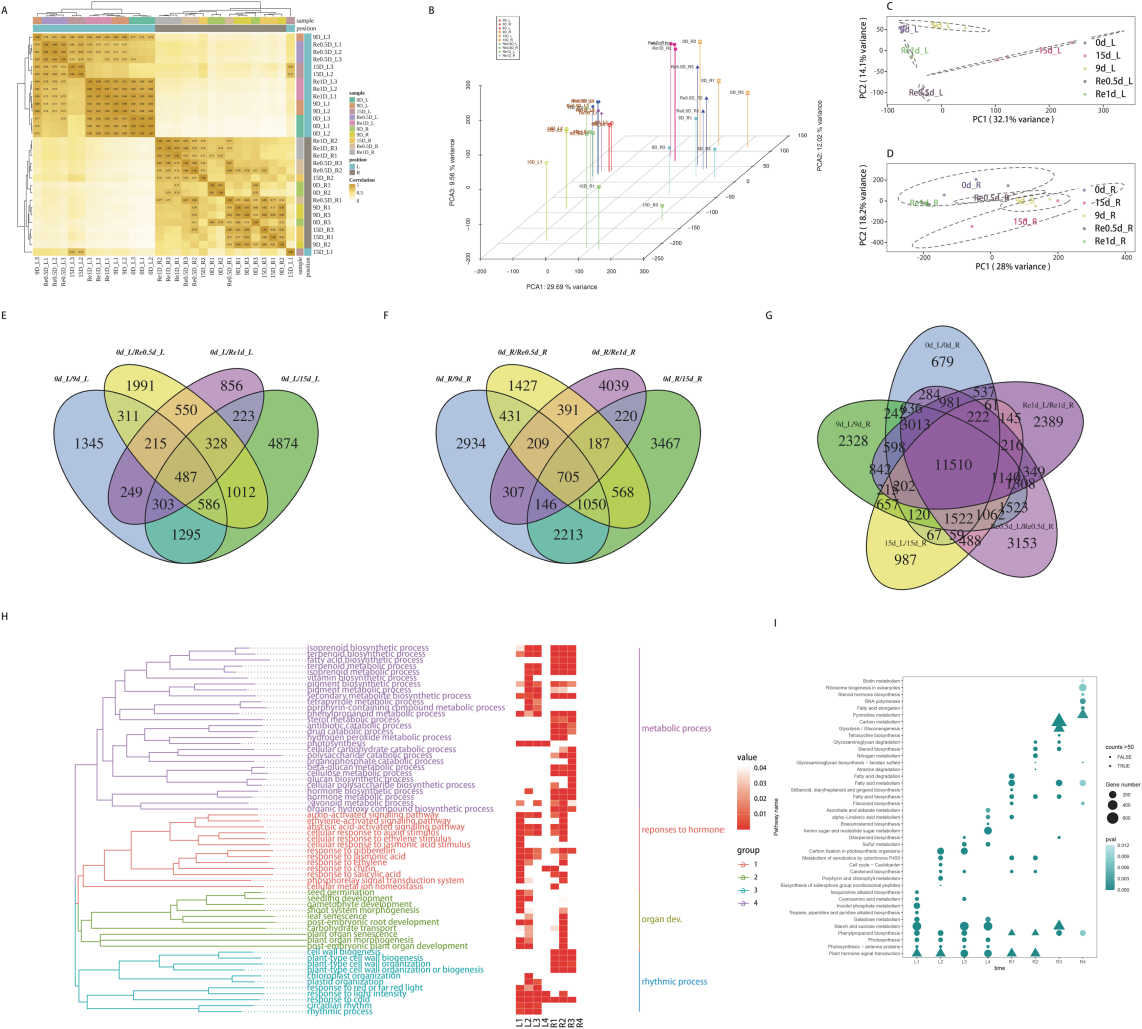
Analysis of transcriptome data and functional enrichment of differentially expressed genes. (A) Heatmap of the correlation of sample gene expression; (B) three-dimensional PCA; (C) leaf PCA; (D) root PCA; (E) Venn diagram of DEGs in different leaf combinations; (F) Venn diagram of DEGs in different root combinations; (G) Venn diagram of DEGs between the leaves and roots at different time periods; (H) GO enrichment similarity clustering and heatmap; (I) KEGG top 10 bubble chart.

The distance clustering heatmap between the samples revealed that the samples were reproducible and the leaf and root tissues were distinct (Fig. 2A). PCA revealed that the repeatability was better among groups and the cumulative contribution rate was high, PC1 on the X axis captures the greatest variation and accounts for 29.69% of the total variance around the PCs whilst PC2 on the Y axis captures the second greatest variation and accounts for 9.56% of the total variance around the PCs, samples of leaves and roots are closely grouped together, which is related to tissue specificity (Fig. 2B). PCA analysis of the leaves (Fig. 2C) and roots (Fig. 2D) revealed that the samples of the leaves in each time group were more clearly separated, which may indicate that the leaves responded more to the drought than the roots. After screening for DEGs, compared to 0d_L (Fig. 2E), the DEGs of the corresponding parts of each stage included 4,791 (9 d), 9,108 (15 d), 5,480 (Re0.5d), and 3,211 (Re1d) DEGs. Compared to 0d_R (Fig. 2F), the DEGs of the corresponding parts of each stage included 7,995 (9 d), 8,556 (15 d), 4,968 (Re0.5d), and 6,204 (Re1d) DEGs. After 15 d, the number of DEGs reached its peak value compared to 0 d. The five differential combinations between the L and R sites had a total difference of 11,510 DEGs (Fig. 2G), which were associated with tissue specificity.

### Functional enrichment analysis of DEGs

The GO similarity enrichment (BP category) revealed that the leaf responses to hormones were active in the L1 group (Fig. 2H). In the L2 group, photosynthesis (ko00195), photosynthesis-antenna proteins (ko00196), and porphyrin and chlorophyl metabolism (ko00860) were the most enriched terms (Fig. 2I). Most of the DEGs in the pathway were downregulated in 15d_L. The light-harvesting complex I chlorophyl a/b binding protein 1-5 (*Lcha1-5*) and *Lhcb1-7* were lowered in 15d_L.

The biosynthesis of metabolites in the roots was enriched in the R2 group (Fig. 2I). In the R2 group, the most significantly enriched pathway included phenylpropanoid biosynthesis (ko00940), and most of the ko00940 DEGs were downregulated in 15d_R. In the R1, R3, and R4 groups, fatty acid biosynthesis (ko00061) and fatty acid metabolism (ko01212) were enriched.

The recovery of plant phenotypes was rapidly developed after rehydration. In the 15d_L/Re0.5d_L group, photosynthesis-related genes were activated, and *Lca1-5* and *Lcb2-6* were upregulated in Re0.5d_L. In the R3 group, fatty acid biosynthesis (ko00061)-related DEGs were all downregulated in Re0.5d.

**Figure 3:**
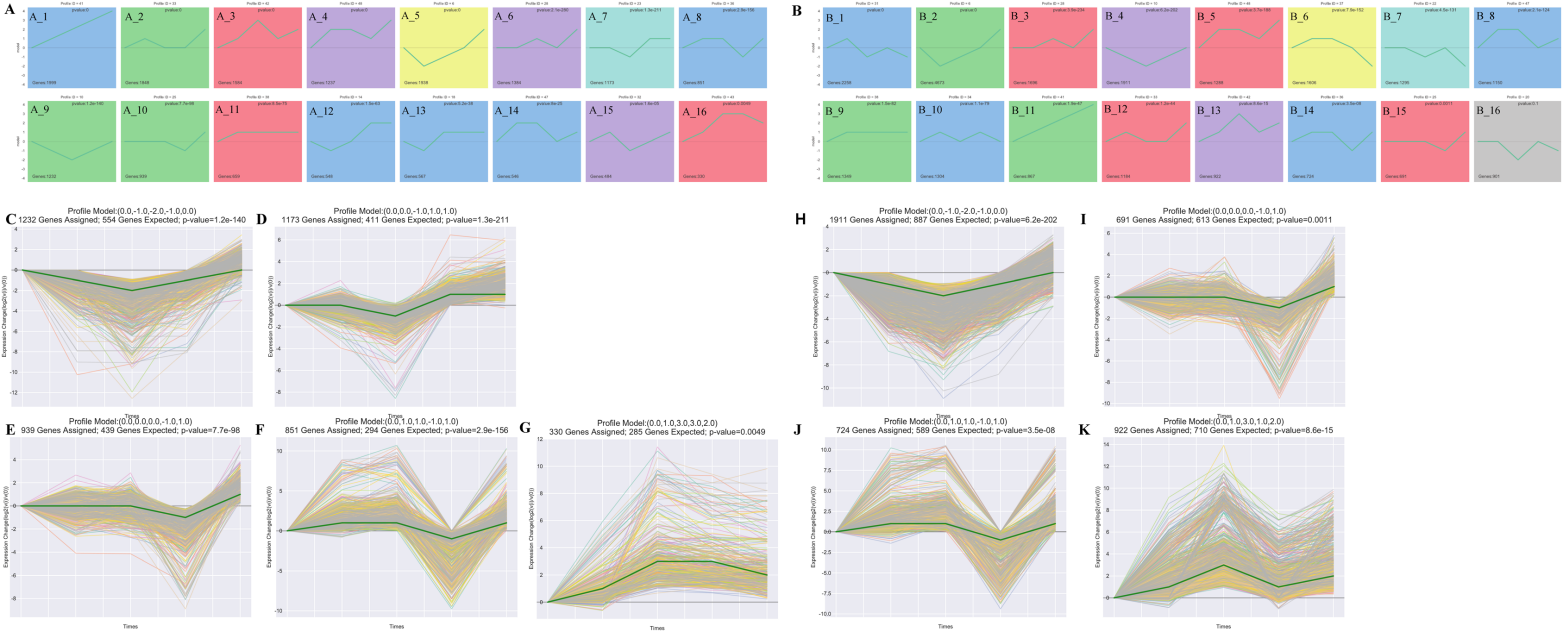
Cluster analysis of the gene expression trends during *T. fortunei* drought. (A) Time-series analysis significance module of the leaves during drought stress; (B) time-series analysis significance module of the roots during drought stress; (C–G) selected significant leaf profiles (C) profile10, (D) profile23, (E) profile25, (F) profile36, (G) profile43; (H–K) selected significant root profiles (H) profile10, (I) profile25, (J) profile36, (K) profile42.

The STEM analysis revealed that there were 16 significantly different modules in the leaf group (Fig. 3A), profile 23 (0.0, 0.0, −1.0, 1.0, 1.0) (Fig. 3D), and profile 10 (0, −1, −2, −1, 0) (Fig. 3C), which all dropped to their lowest values after 15 d. In profile10, photosynthesis (ko00195), photosynthesis-antenna proteins (ko00196), carbon fixation in photo, and synthetic organisms (ko00710) were enriched. Profile43 (0.0, 1.0, 3.0, 3.0, 2.0)-related gene expression increased to its maximum value after 15 d (Fig. 3G). Starch and sucrose metabolism (ko00500) and diterpenoid biosynthesis (ko00904) were enriched in Profile43. Profile25 (0.0, 0.0, 0.0, −1.0, 1.0) and profile 36 (0.0, 1.0, 1.0, −1.0, 1.0)-related gene expression were reduced to their lowest values in Re0.5d (Figs. 3E and 3F). Arachidonic acid metabolism (ko00590) and linoleic acid metabolism (ko00591) were enriched in profile25. Fatty acid biosynthesis (ko00061) and fatty acid elongation (ko00062) were enriched in profile36.

There were 15 significant differences in the root group (Fig. 3B). Profile10 (0.0, −1.0, −2.0, −1.0, 0.0)-related gene expression reached its lowest value after 15 d (Fig. 3H). Phenylpropanoid biosynthesis (ko00940) and glycosaminoglycan degradation (ko00531) were enriched in Profile10. Profile42 (0.0, 1.0, 3.0, 1.0, 2.0)-related gene expression peaked after 15 d (Fig. 3K), at which point, linoleic acid metabolism (ko00591), cutin, suberine, and wax biosynthesis (ko00073), and sulfur metabolism (ko00920) were enriched. In Profile25 (0.0, 0.0, 0.0, −1.0, 1.0), fatty acid biosynthesis (ko00061) was enriched (Fig. 3I). Profile36 (0.0, 1.0, 1.0, −1.0, 1.0)-related gene expression reached its lowest values in Re0.5d (Fig. 3J). Starch and sucrose metabolism (ko00500) and galactose metabolism (ko00052) were also enriched in Profile36.

### Weighted correlation network analysis

**Figure 4:**
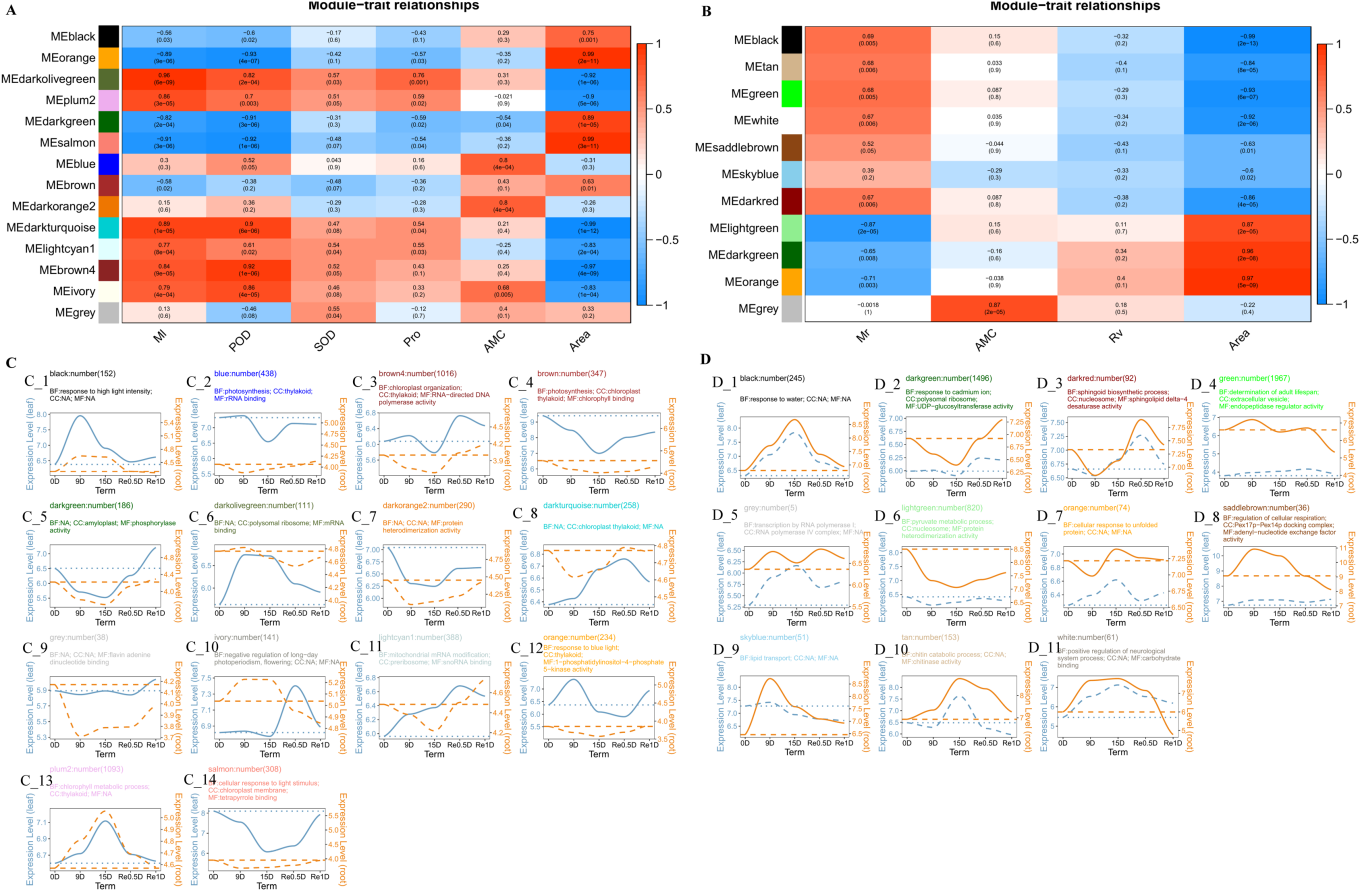
Analysis of the co-expression networks. (A) Leaf trait and module correlation map; (B) different module gene expression patterns in the leave; (C) root trait and module correlation map; (D) different module gene expression patterns in the roots. Note: B and D represent the expression trend of genes in the lower leaves and roots of different modules (the average of all genes in the module was normalized and linearly fitted); the left of the double axis is the tick mark (“# 6B9EC2”) that represents the leaves; the right of the double axis is the tick mark (“# EE861A”) that represents the roots; the text includes the number of gene modules, module names, and GO enrichment analysis results (displayed with the smallest p-value for each of BP, CC, and MF category); The gene trends in the module are processed by taking the logarithm of the gene expression in the module, and fitted in a linear simulation.

The Weighted correlation network analysis (WGCNA) revealed that the leaves divided into 14 modules and the roots divided into 11 modules. In the leaf co-expression analysis network (Fig. 4A), the lightcyan1 modeules was significantly positively correlated with Ml (Correlation coefficient = 0.77, *P* = 8e−04), darkturquoise modules was significantly positively correlated with Ml (Correlation coefficient = 0.89, *P* = 1e−05) and POD (Correlation coefficient = 0.9, *P* = 6e−06), the black module was significantly negatively correlated with Ml (Correlation coefficient = −0.56, *P* = 0.03), and the brown4 module was correlated with rehydration stress expression after 15 d in Re0.5d (Fig. 4B). The GO enrichment analysis (CC category) revealed that the brown4 module was associated with thylakoid (GO:0009579) and chloroplast thylakoid (GO:0009534) (Fig. 4C_3). The brown module was significantly positively correlated with area (Correlation coefficient = 0.63, *P* = 0.01). Additionally, the GO enrichment analysis (CC category) indicated that chloroplast thylakoid and plastid thylakoid (GO:0031974) were enriched (Fig. 4C_4). The darkorange2 module and AMC were significantly positively correlated (Correlation coefficient = 0.8, *P* = 4e−04), protein heterodimerization activity (GO:0046982) was enriched (MF category) (Fig. 4C_7).

In the root co-expression analysis network (Fig. 4B), the black module was significantly negatively correlated with area (Correlation coefficient = −0.99, *P* = 2e−13). Response to water (GO:0009415), response to cold (GO:0009409), and response to salt stress (GO:0009651) (BP category) were enriched (Fig. 4D_1). *DHNs COR410-like* (TRINITY_DN51483_c1_g1_i2), *LEA14* (TRINITY_DN41505_c0_g2_i2), *NAC 47* (TRINITY_DN48903_c0_g2_i1), and *Annexin D2* (TRINITY_DN46177_c1_g1_i1) aggregated in response to water (GO:0009415). The light green module was significantly positively correlated with area (Correlation coefficient = 0.87, *P* = 2e−05). Protein heterodimerization activity (GO:0046982), water transmembrane transporter activity (GO:0005372), and water channel activity (GO:0015250) (CC category) were enriched (MF category) (Fig. 4D_6).

### Real-time quantitative PCR results

**Figure 5:**
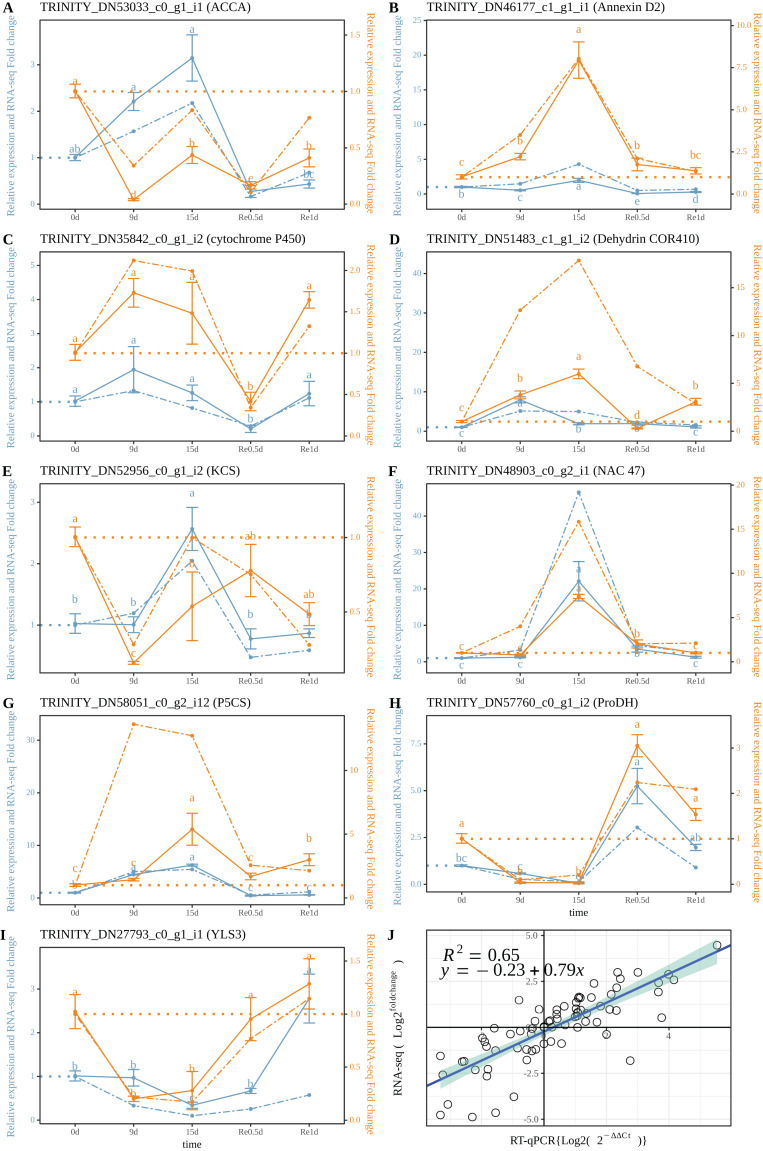
Correlation between the RT-qPCR and RNA-Seq data. (A–I) RT-PCR expression of a selected gene; (J) Correlation between the RT-qPCR and RNA-Seq. Data are from three biological replicates and three technical replicates. The expression analysis was conducted based on the 2^−ΔΔct^ method. The left of the double axis is the tick mark (“# 6B9EC2”) that represents the leaves; the right of the double axis is the tick mark (“# EE861A”) that represents the roots; the solid line represents RT-qPCR; the dashed line represents the fold change. Datas were evaluated by one-way ANOVA followed by LSD post hoc tests, performed by R.

The RT-qPCR results revealed that TRINITY_DN53033_c0_g1_i1 (*ACCA*, Fig. 5A) in roots and leaves showed a significant down-regulated expression at Re0.5d (Compared to 0d); TRINITY_DN46177_c1_g1_i1 (*Annexin D2*, Fig. 5B), TRINITY_DN51483_c0_g1_i2 (*Dehydrin COR410*, Fig. 5D), TRINITY_DN48903_c0_g2_i1 (*NAC47*, Fig. 5F) showed up-regulated expression in roots at 15d (Compared to 0d) Log2 (^Fold change^) and log2 (2^−ΔΔCt^) were subjected to linear correlation analyses (Fig. 5J). Results revealed a positive correlation (0.79) with the RNA-Seq results (*R*^2^ = 0.65). Thus, the RT-qPCR and RNA-Seq data were relatively consistent.

## Discussion

Drought is one of the main abiotic stressors affecting plant growth and development. Drought responses may vary in different plant organs or tissue and the analysis in both mature leaves and root tips is critical for plants to adapt to water deficits (Feng et al., 2017). After entering the drought state, *T. fortunei* leaves fold, root activity continuously decreases, Pro activity continuously increases, and the genes related to photosynthesis (ko00195) and photosynthesis-antenna proteins (ko00196) are continuously downregulated (0, −1, −2, −1, 0). Under drought stress, the light absorption of antenna proteins decreases, electron transport rates of PSII and PSI decreases, ROS accumulation increases, photosynthetic pigments are destroyed, *RAC* activity decreases (Din et al., 2011; Sharma et al., 2012; Guo et al., 2017; Dalal & Tripathy, 2018; Luo et al., 2018; Michaletti et al., 2018). Resuscitation plants are able to survive 95% of their cell water loss, one tolerance mechanism is to reversibly shut down photosynthesis, for example, in *Xerophyta humilis*, *psbR*, *psbA*, and *psbP* were downregulated during dehydration, and complex water regulation expression trends were exhibited (Dace et al., 1998; Collett et al., 2003). In this study, under drought stress, the expression of chlorophyl a-b binding proteins decreased and the synthesis of photosynthesis-related factors decreased. After rehydration, *RAC* and other photosynthetic-related genes were activated and recovered to a relatively consistent level after 0 d in Re1d. Gene expression of the thylakoid-associated cellular components proliferated after rehydration (Fig. 4C_3, brown4). *T. fortunei Psb O*, *Psb P, Psb Q, Psb R*, and other PSII subunits were downregulated under drought stress and gradually increased after rehydration. *T. fortunei* also reduced its light-trapping ability and the composition of the photosynthetic apparatus, thereby reducing photosynthesis and increasing drought resistance by leaf folding to prevent light-induced chloroplast ROS damage due to dehydration.

Plant roots may induce specific stress responses to cope with the early perception of soil water loss (Haas et al., 2019). MDA is a product of membrane lipid peroxidation that is produced when plants are under stress, and the content of MDA can reflect the degree of cell membrane damage (Dong et al., 2019). The MDA content in the root reaches its peak at 12d, which is faster than the time node of the highest MDA value in the leaf (Figs. 1F and 1G) In the R2 group, phenylpropanoid biosynthesis (ko00940) was curbed, most of the DEGs (48/53) were downregulated in 15d_R, and some genes (27) of profile10 (0.0, −1.0, −2.0, −1.0, 0.0) were expressed. Protein phosphorylation and dephosphorylation are important signaling events that lead to drought tolerance (Deeba, Pandey & Pandey, 2016). ABA signaling is majorly composed of three core components: ABA receptor, protein kinases, and protein phosphatases (Saini, Singh & Pandey, 2020). PP2C belongs to a group of phosphatases involved in ABA signaling and is a negative regulator (Zhang & Gan, 2012). After ABA, polyethylene glycol and dehydration treatments, the accumulation rate of *OsPP2C09* (*Oryza sativa* L.) transcripts in roots was more rapid and greater than that in shoots (Zhang et al., 2020). Both PP2C-related DEGs in L2 and R2 groups were upregulated after 15 d. In order to absorb water and survive under drought stress, various organic solutes accumulate in the cytoplasm and chloroplasts for osmotic adjustment (Rajasheker et al., 2019). ABA can induce the accumulation of intracellular osmoprotectants, such as the LEA post-embryonic protein, chaperone proteins, carbohydrates, and Pro, which may be critical for survival under drought stress (Wang et al., 2018). The relationship between water transmembrane transporter activity (GO:0005372) and water channel activity (GO:0015250) was positively correlated with area (Fig. 4D_6, lightgreen), the black module was significantly negatively correlated (Fig. 4B), and the expression of DHNs was generally regulated and induced by ABA, which can reduce root water conductivity (Choi, Zhu & Close, 1999; Hand et al., 2011; Li et al., 2019a). LEA proteins bind to a large number of water molecules and maintain normal metabolism in cells (Ingram & Bartels, 1996). In a previous study, rice *OsANN3* was found to mediate Ca^2+^ influx by binding to phospholipids, and overexpression significantly increased drought stress survival (Li et al., 2019b). *DHNs COR410-like* (TRINITY_DN51483_c1_g1_i2), *LEA14* (TRINITY_DN41505_c0_g2_i2), *NAC 47* (TRINITY_DN48903_c0_g2_i1), and *Annexin D2* (TRINITY_DN46177_c1_g1_i1) aggregated in response to water (GO:0009415) term which is enriched in root black modules (Fig. 4D_1). qRT-PCR showed *Annexin D2* (Fig. 5B), *Dehydrin COR410* (Fig. 5D), *NAC47* (Fig. 5F) upregulated in roots at 15d. As drought persisted, phenylpropanoid biosynthesis in the roots was suppressed, the *DHNs*, *LEA*, *Annexin D2*, *NAC*, and other genes were expressed, possibly to protect cell membrane permeability in *T. fortunei* root tissues.

Resilience is an important physiological feature of drought-tolerant genotypes. The ability to preserve tissue health, integrity, and avoid aging is vitally important. Resurrection plants may have pathways that inhibit drought-related senescence (Rivas et al., 2016; Challabathula, Zhang & Bartels, 2018; Griffiths, Gaff & Neale, 2014). The biosynthesis of palmitic (C16:0), linoleic (C18:2), linolenic (C18:3) and stearic acid (C18:0) increased 18, 12, 20, and 8-fold during dehydration in *Pleopeltis polypodioides*, rehydration lowered levels of peroxides, the activity of glutathione-oxidizing enzymes, and fatty acids (John & Hasenstein, 2018). After *T. fortunei* rehydrated for 12 h, the leaves gradually recovered from the fully folded state. In profile36 (0.0, 1.0, 1.0, −1.0, 1.0), fatty acid biosynthesis (ko00061) (TRINITY_DN53033_c0_g1_i1; TRINITY_DN67658_c0_g1_i1) was enriched in the leaves. Meanwhile, in root profile25 (0.0, 0.0, 0.0, −1.0, 1.0) (Fig. 3B), the fatty acid biosynthesis (ko00061) (TRINITY_DN48458_c0_g1_i1; TRINITY_DN52213_c0_g1_i2; TRINITY_DN53033_c0_g1_i1) pathway was simultaneously enriched. The transcripts of fatty acid biosynthesis (TRINITY_DN53033_c0_g1_i1; TRINITY_DN67658_c0_g1_i1) were annotated as ACCase subunit alpha (acetyl-coenzyme A carboxylase carboxyl transferase subunit alpha, chloroplastic), whose transcripts (TRINITY_DN52213_c0_g1_i2) encoded stearoyl-ACP desaturase (SAD). ACCase is a key, rate-limiting enzyme involved in fatty acid biosynthesis that catalyzes the carboxylation of acetyl-CoA to form malonyl-CoA, providing a substrate for the synthesis of fatty acids and many secondary metabolites (Rajasekharan & Nachiappan, 2010). Fatty acid is the main component of cell and organelle membrane lipids. The regulation of membrane lipid contents and compositions is an effective regulation method for adapting to drought conditions, as well as a positive regulation mechanism during recovery after rehydration (Liu & Tingting, 2018). In the R3 group, fatty acid biosynthesis (ko00061)-related DEGs were downregulated in Re0.5d and some genes in profile25 (0.0, 0.0, 0.0, −1.0, 1.0) were expressed in the roots. Additionally, ɑ-CT reached its lowest expression level in Re0.5d. This indicated that fatty acid biosynthesis in *T. fortunei* roots is repressed during the rehydration phase after extreme drought.

## Conclusions

Under drought stress, *T. fortunei* reduces its light-trapping ability and the composition of the photosynthetic apparatus, thereby reducing photosynthesis and increasing drought resistance by leaf folding to prevent light-induced chloroplast ROS damage to dehydration. As drought conditions worsen, phenylpropanoid biosynthesis in the roots is suppressed, *DHNs*, *LEA*, *Annexin D2*, *NAC*, and other genes that may protect the cellular membrane’s permeability in *T. fortunei* root tissues. Fatty acid biosynthesis in *T. fortunei* roots is repressed after rehydration.

## Supplemental Information

10.7717/peerj.10933/supp-1 Supplemental Information 1 Raw data.

## Additional Information and Declarations

### Data Availability

The following information was supplied regarding data availability: Data is available as a Supplemental File and at NCBI: PRJNA598974.
